# Auricular Point Acupressure to Manage Chronic Low Back Pain in Older Adults: A Randomized Controlled Pilot Study

**DOI:** 10.1155/2014/375173

**Published:** 2014-07-24

**Authors:** Chao Hsing Yeh, Natalia E. Morone, Lung-Chang Chien, Yuling Cao, Huijuan Lu, Juan Shen, Leah Margolis, Shreya Bhatnagar, Samuel Hoffman, Zhan Liang, Ronald M. Glick, Lorna Kwai-Ping Suen

**Affiliations:** ^1^School of Nursing, University of Pittsburgh, 3500 Victoria Street, 440 Victoria Building, Pittsburgh, PA 15261, USA; ^2^Department of Medicine, Division of General Internal Medicine, University of Pittsburgh, School of Medicine, Veterans Administration, Pittsburgh Healthcare System, Geriatric Research, Education and Clinical Center, 230 McKee Place Suite 600, Pittsburgh, PA 15213, USA; ^3^Division of Biostatistics, University of Texas, School of Public Health at San Antonio Regional Campus and Research to Advance Community Health Center, University of Texas Health Science Center at San Antonio Regional Campus, 7411 John Smith Drive, Suite 1050 Room 505, San Antonio, TX 78229, USA; ^4^School of Nursing, Fudan University, 305 Fenglin Road, Shanghai, China; ^5^School of Nursing, Suzhou Health College, No. 28 Kehua Road Northern District of Suzhou International Education Park, Suzhou, China; ^6^Departments of Psychiatry, Physical Medicine and Rehabilitation, School of Medicine, University of Pittsburgh, 3811 O'Hara Street, Pittsburgh, PA 15213, USA; ^7^School of Nursing, The Hong Kong Polytechnic University, Hung Hom, Kowloon, Hong Kong

## Abstract

This prospective, randomized clinical trial (RCT) pilot study was designed to (1) assess the feasibility and tolerability of an easily administered, auricular point acupressure (APA) intervention and (2) provide an initial assessment of effect size as compared to a sham treatment. Thirty-seven subjects were randomized to receive either the real or sham APA treatment. All participants were treated once a week for 4 weeks. Self-report measures were obtained at baseline, weekly during treatment, at end-of-intervention (EOI), and at a 1-month follow-up. A dropout rate of 26% in the real APA group and 50% in the sham group was observed. The reduction in worst pain from baseline to EOI was 41% for the real and 5% for the sham group with a Cohen's effect size of 1.22 (*P* < 0.00). Disability scores on the Roland Morris Disability Questionnaire (RMDQ) decreased in the real group by 29% and were unchanged in the sham group (+3%) (*P* < 0.00). Given the high dropout rate, results must be interpreted with caution; nevertheless, our results suggest that APA may provide an inexpensive and effective complementary approach for the management of back pain in older adults, and further study is warranted.

## 1. Introduction

Chronic low back pain (CLBP) is the most common self-reported physician-diagnosed pain condition among those 65 years or older in the United States [1–3]. Additionally, CLBP imposes a significant societal and economic burden on the U.S. healthcare system [4–6]. Multidisciplinary approaches (i.e., education, exercise, analgesics, spinal manipulation, and behavior) have suggested protocols, techniques, or guidelines for acute and chronic back pain [7], but these treatments have limited efficacy [8]. Moreover, older adults are less likely to receive adjunctive care for their pain such as spinal manipulation, massage therapy, or electrical stimulation [8, 9]. The continued high prevalence of CLBP highlights the need for better pain management strategies.

Analgesic use with nonsteroidal anti-inflammatory medications or opioids is one of most common strategies for managing CLBP [10], but it is associated with a variety of adverse side effects, which include drowsiness, constipation, dry mouth, gastrointestinal bleeding, and potential for addiction [11, 12]. For these reasons, improved nonpharmacological pain management is needed. Acupuncture, one of most commonly used complementary and alternative medicines (CAM), has been suggested as an adjunctive therapy for CLBP [13]. As one form of CAM, acupuncture is already used by a large and growing number of individuals in the United States, and musculoskeletal conditions and pain are the top reasons for the use of acupuncture [14]. In a 2007 government survey, more than 3 million American adults had not only used acupuncture for back pain over the previous 12 months [15], but also spent an estimated $33.9 billion out-of-pocket on CAM and $11.9 billion on visits to CAM practitioners, including acupuncturists [16]. A more widespread application of acupuncture to manage pain is restricted by the need for patients to travel frequently to receive acupuncture treatment (i.e., two sessions per week for four weeks, and then weekly for four weeks) [17] and the cost of the acupuncture treatments that are not covered by insurance [16, 18, 19].

Auricular therapy, a variant of acupuncture rooted in traditional Chinese medicine (TCM), uses noninvasive acupoints on specific areas of the outer ear to treat disease/illness [20, 21]. In TCM, a disease is caused by the imbalance of a person's energy or* qi* [20]. The stimulation of auricular acupoints regulates* qi*, activates the energy pathways (meridians) and collateral systems, and has, therefore, been successful in treating health problems [20]. In 1950, Dr. Paul Nogier, a French neurosurgeon, theorized that the outer ear represents an inverted fetus within the womb and, thus, proposed the somatotopic correspondence of specific parts of the body to specific parts of the ear [22]. The current auricular therapy practiced worldwide is based on Nogier's principles.

The World Health Organization considers auricular medicine a form of microacupuncture that can affect the whole body [23]. Although popular in Asia for over 2,000 years and in Europe for the past 60 years, it has not yet become widely practiced by health care providers in the United States. Despite this, auricular therapy has shown some promising results for pain management [24–29]. A recent meta-analysis of auricular therapy for pain management reports that auricular therapy can not only reduce analgesic use for perioperative pain, but also reduce pain intensity for acute and chronic pain [24]. The meta-analysis of auricular therapy included acupuncture, electroacupuncture, and acupressure among 17 studies: 8 for perioperative pain, 4 for acute pain, and 5 for chronic pain. The authors of the meta-analysis conclude that auricular therapy may be used as an adjunct therapy for pain management [24]. However, the evidence supporting auricular therapy remains limited: few studies have been RCTs, sample sizes have tended to be small, blinding procedures often have been inadequate, and several studies have lacked a sham group comparison [24]. Therefore, the clinical effectiveness of auricular therapy remains difficult to assess.

Auricular therapy can be administered via auricular acupuncture, electroacupuncture, and acupressure. In contrast to auricular acupuncture and electroacupuncture, which need to be practiced by licensed practitioners, auricular point acupressure (APA) applies an acupuncture-like stimulation to specific points on the ear (acupoints) without using a needle. The stimulation is provided using small, spherical* Vaccaria* seeds placed on the outer ear with small pieces of adhesive tape. Once the seeds are taped to the ear by a trained therapist (e.g., nurse), patients can stimulate the acupoints by pressing them as directed to achieve acupuncture-like effects. Through this self-management, fewer visits to a therapist are required. Our pilot data on a younger population with CLBP showed that APA provided immediate pain relief (40% reduction in pain intensity 1 day after APA) [27]. Our 4-week APA treatment achieved even greater pain relief (75% pain reduction), physical function improvement (42%), and maintained such effects at one-month follow-up [28].

Given this promising preliminary evidence of the effects of APA for pain relief, improved physical function, reduced analgesic use, and reduced cost, we decided to pilot test APA for CLBP among individuals in an older population seeking pain relief. Catastrophizing may increase macrophage activation and, thus, inflammation, as suggested by a study of laboratory-induced pain in healthy volunteers [30]. The fear-avoidance model of pain posits that catastrophizing leads to fear of movement (kinesiophobia) [31]. Older adults who have a lower level of fear-avoidance related to pain have a lower level of disability [32]. This study assessed (1) the feasibility of recruiting CLPB patients who are older adults, (2) the adherence safety/tolerability (i.e., somatic symptoms), and (3) outcomes with an APA intervention for CLBP among this group.

## 2. Methods

### 2.1. Overview

This study was approved by the University of Pittsburgh (Pitt; approval number PRO10010063). Participant eligibility was screened over the phone. After informed consent and baseline assessments were obtained, participants were randomized into either the intervention (real APA) or placebo (sham APA) group with a computer generated randomization routine with blocks of length 4. The intervention included four in-person sessions over 4 weeks. Real APA consisted of treatment based on genuine acupressure points for CLBP. Sham APA consisted of treatment with acupressure points not correlated with CLBP. Over the duration of the 4-week treatment, participants received weekly seed placement, left the taped seeds on their ears for 5 days, and then removed the seeds for 2 days before a new treatment began. The study outcomes and blood samples for inflammatory markers were assessed at baseline, weekly during APA treatment, at end-of-intervention (EOI), and 1-month follow-up. All participants received free parking during the treatment and $50.00 after completion of the study assessment.

### 2.2. Participants and Setting

Participants who were recruited for this feasibility study were included if they met the following criteria: (1) aged 65 years or over, (2) intensity of pain ≥4 on a 10-point numerical pain scale, (3) had low back pain for at least 3 months and lower back pain intensity greater than that of any other body part, (4) were willing to commit to weekly study visits for 4 weeks and then two follow-up visits (at EOI and at 1 month thereafter), and (5) were able to read and write English. Participants were excluded from the study if they had (1) an inflammatory, malignant, or autoimmune disease, (2) a compression fracture caused by osteoporosis, spinal stenosis, spondylolysis, or spondylolisthesis as these conditions might be confound treatment effects or interpretation of results (e.g., severe fibromyalgia and rheumatoid arthritis), or (3) an allergy to the tape for the seeds. Participants were recruited by (1) flyers in primary care offices and clinics placed at UPMC and (2) participant referrals from the coauthor's (NM) study participants who completed the mind and body intervention for CLBP. The Clinical and Translational Science Institute (CTSI) Research Participant Registry at Pitt was also used to recruit potential participants. Potential participants who expressed interest in the study were contacted by the study coordinator by phone who explained the study in detail, screened the potential participants for eligibility, and scheduled a clinic visit if possible. The study was conducted at the Pitt School of Nursing, Clinical Research Suite.

### 2.3. APA Treatment Protocol

**Figure 1:**
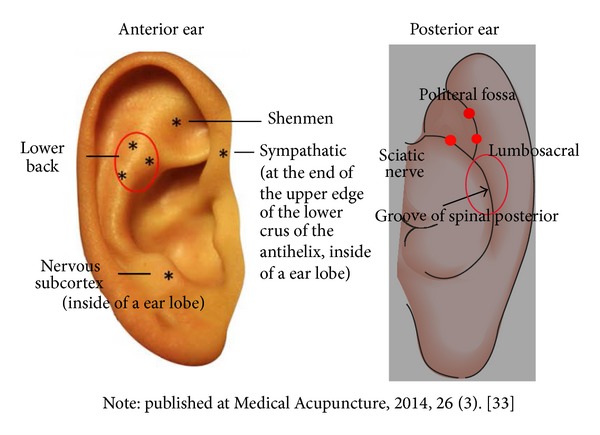
Ear acupoints selected for treatment.

In order to ensure the accuracy of acupoint selection, the following procedures were used. (1) The three points for alleviating stress and pain (i.e., shenmen (master points for sedation), sympathetic (to alleviate stress and pain), and nervous subcortex (to alleviate stress and pain)) according to disharmony of zang organs and disturbance of meridians are located on the anterior ear. (2) The active points corresponding to CLBP (appearing as the waist triangle cluster) and the grooves of the spinal and sciatic posterior are all located on the posterior side of the ear (see Figure 1) [33]. An electrical point finder (manufactured by AMIRTC) was used to locate the points, which is done by detecting decreased resistance.* Vaccaria* seeds were placed on the subject based on the results of the AMIRCT. Control subjects had* Vaccaria* seeds taped onto the stomach, mouth, duodenum, and eye acupoints. Bilateral auricular points were identified for treatment.* Vaccaria* seeds (natural, nontoxic, botanical seeds), approximately 2 mm in diameter, were applied to the ears and held in place with water-resistant tape.

Participants were told to press the seeds on each ear at least 3 times a day for 3 minutes each time. Participants were also told to press the seeds whenever they had pain and to remove the tape and seeds after 5 days. Doing so ensured that not only the ear was free of tape 2 days each week to minimize the risk of an allergic reaction to the tape, but also the acupoints were allowed time to recover and restore sensitivity prior to the next treatment. Each participant was given a diary to monitor their adherence to the treatment protocol (the actual times they press the seeds each day and the duration of applied pressure), analgesic use, related medication and supplements, and pain intensity. All data were collected by a trained collector who was blinded to the group assignment of the participants.

### 2.4. Measures

Table 1 lists the study measures used in the study, including the number of items in each scale, score range, and internal consistency. All measures were collected at baseline, weekly for 4 weeks during the APA treatment, and 1 month after the conclusion of the APA treatment. Internal consistency (Cronbach's *α*) [34] for each measure was acceptable.

#### 2.4.1. Primary Outcomes

Primary outcomes included worst pain and physical functioning. “Worst Pain” was an individual item from the brief pain inventory short form (BPI-sf) [35]. The cut-off point of 10–20% is rated as “minimally important,” ≥30% as “moderately important,” and ≥50% as “substantial” pain intensity change from baseline on a 0–10 numerical scale [36].* Physical Functioning* was measured by the Roland Morris Disability Questionnaire (RMDQ) [37]. The RMDQ is a 24-item measure to assess the impact of back-related pain on daily functioning. Participants were asked to select “yes” or “no” for statements related to their physical function. The total score ranged from 0 (no disability) to 24 (maximum disability). RMDQ is a reliable, valid, and sensitive measure that demonstrated substantial construct validity [37, 38]. A reduction of RMDQ 30% or greater is rated as “minimally clinically important” [39].

#### 2.4.2. Other Outcomes

*(1) Pain Quality.* The Short Form McGill Pain Questionnaire (MPQ-SF) [40] is a self-reported measure to assess the quality and intensity of pain. MPQ-SF consists of 15 descriptors (11 sensory and 4 affective) on an intensity scale defined as follows: 0 = none; 1 = mild; 2 = moderate; and 3 = severe. Three pain rating indices (PRI) were used for final data analysis, including sensory, affective, and total.

*(2) Other Dimensions Related to Pain*. Pain was additionally measured by two subscales of “Affective Distress” (AD—2 items) and “Life Control” (LC—2 items) related to pain from the multidimensional pain inventory screening (MPI-s) [41]. Higher scores for these dimensions indicated more severity within the subscales.

*(3) Emotional Functioning*. This was measured with the generalized anxiety disorder 7 (GAD-7) [42] scale and two subscales of “Anxiety” and “Depression” from the Patient-Reported Outcomes Measurement Information System (PROMIS) Short Form [43, 44]. The GAD is a self-reported questionnaire for the screening and severity of generalized anxiety disorder with seven items, which measure severity of various signs of generalized anxiety disorder according to reported response categories of “not at all,” “several days,” “more than half the days,” and “nearly every day.” The PROMIS is an NIH Roadmap initiative designed to improve self-reported outcomes (http://www.nihpromis.org). The subscales of “depression” (8-item) [44] and “anxiety” (8-item) [44] were used to assess the participants' symptoms during the past 7 days.

*(4) Pain Belief*. The Fear Avoidance Beliefs Questionnaire (FABQ) [42] is a 16-item, self-report scale that focuses specifically on a patient's beliefs about how physical activity and work affects his or her pain. Four items were selected from the physical activity factor to form a modified version (mFABQ). In a pilot testing with 36 acute back pain sufferers, this four-question mFABQ was found to be highly correlated with the five-question physical activity scale of the FABQ (*r* = 0.47) [42].

*(5) Catastrophizing*. The pain and catastrophizing scale (PCS) [45] was used in order to measure exaggerated and negative interpretations of pain. The PCS was a self-report scale that consists of 13 items. Participants were asked to reflect on past painful experiences and to indicate to which degree he or she experienced each of the following when feeling pain: rumination (4 items), magnification (3 items), or helplessness (6 items). The PCS features a 0–4 Likert scale (score sum 0–52) from “not at all” to “all the time.” A higher PCS score indicates stronger catastrophizing.

*(6) Health Related Quality of Life. *The WHO Quality of Life-BREF (WHOQOL-BREF) [46] was used to measure quality of life. The WHOQOL-BREF includes 26 items, is self-administered, and measures the following broad domains: physical health, psychological health, social relationships, and environment. It was derived from the WHOQOL-100, and this 26-item version has established good reliability and validity [46].

*(7) Demographic Data.* A demographic questionnaire was used to collect information on age, marital status, educational level, living arrangement, ethnicity, disease diagnosis, chronic conditions, medication use, and treatment related to CLBP.

*(8) Daily Diary*. A daily diary was given to participants to record their APA self-treatment (including frequency and duration of pressing the taped-on seeds and any side effects), medication use (including supplements), and three items of pain intensity from the BPI-sf (worst, average, and current). Due to word limitations, data from the daily diary are not presented in this manuscript.

### 2.5. Data Analysis

In order to examine the true effects of the APA, two types of analyses were conducted for primary outcomes: intent-to-treat (ITT) analysis using the data of all participants, and the per-protocol (PP) analysis, which included only those participants who adhered to the APA. The findings of ITT and PP were so similar that only ITT is included for discussion in this paper. Missing values of the outcome variables were replaced by “last value carried forward” for ITT. Descriptive statistics were used to present demographic characteristics and study measures. The equality of the mean change score from baseline to EOI and 1-month follow-up for real and sham groups was tested with the two sample unpooled-*t* test [47]. Cohen's d was used to calculate effect sizes [48].

The adherence rate was defined by the number of participants who were able to follow at least two-thirds of the suggested pressing time (at least 2 times/day, 2 minutes/time) to determine the feasibility of participants practicing APA at home. We applied a Chi-square test [49] to test the difference of proportion for clinical improvement in pain intensity and back-disability between real and sham groups. Becuase this was a pilot study with small sample size, multiple testing correction was not used. Differences were considered statistically significant at the *P* < 0.05 level. All of the data analyses were performed using SAS software, version 9.2 [50].

## 3. Results

### 3.1. Characteristics of the Participants

**Figure 2:**
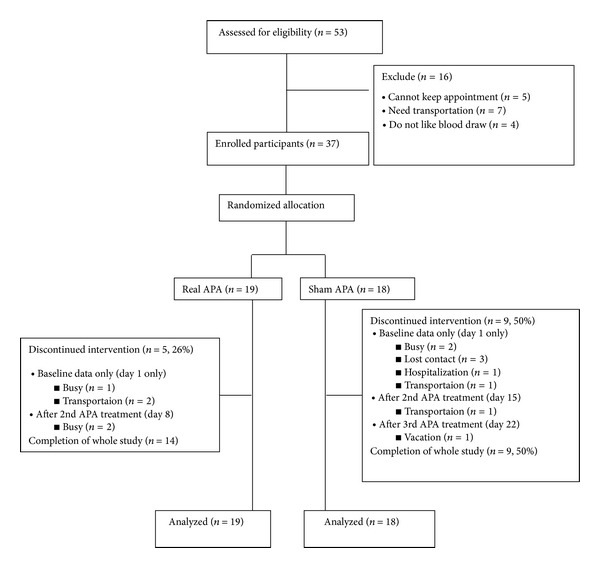
Flow chart of participant recruitment.

Figure 2 shows the flow of participant recruitment. Fifty-three participants contacted the study coordinator to express their interest in the study from October 2012 to June 2013. Sixteen participants were excluded because they were not able to keep a study appointment (*n* = 5), needed transportation (*n* = 7), or refused the blood draw (*n* = 4). At least three reminder calls were made until we ascertained why a participant missed his or her follow-up appointment. In the end, 37 participants were randomized to the real APA (*n* = 19) or sham APA (*n* = 18) group using a computer-generated randomization routine.

Table 2 presents the demographic characteristics of participants. The average age of the participants was 70.6 years (SD = 4.67) in the real APA group and 76.7 (SD = 7.00) in the sham APA group. There were no statistically significant differences among the characteristics of both groups, except for age. Age was used to determine if there were differences for baseline assessment, but no statistical significance was found. We further examined the reasons for and the timeline of dropout among the participants who failed to complete the treatment. We found that participants tended to drop out from the study after the first office visit (*n* = 3 [60%] in real APA and *n* = 7 [78%] in sham APA). Thirty-three (90%) participants believed that they were enrolled in the real APA group.

### 3.2. The Feasibility for Participants to Practice APA

The results indicate that the adherence rate during the 4-week APA was high in week 1 and then gradually decreased. Participants exhibited an 85% or greater adherence rate throughout the 4-week APA for both the real and sham APA groups.

### 3.3. The Safety of APA Treatment

Table 3 presents the adverse effects of APA that were minimal and bearable as reported by the participants. Participants in both groups reported experiencing sensitivity (*n* = 3, 16%), soreness (*n* = 4, 21%), and discomfort (*n* = 4, 21%) of the ears after seed placement. This discomfort usually appeared on day 1 or 2 and then gradually diminished. Participants also reported itching on the ear (*n* = 7, 37%) and sleep disturbance when sleeping on the side that was being treated with APA (*n* = 2, 11%). Participants reported that compared to their back pain, the ear discomfort was tolerable.

### 3.4. Primary Outcomes of the APA Treatment (between Group Comparisons)

The primary outcomes for the real and sham APA groups are presented in Table 4 for ITT and PP. Due to the similar findings (no statistically significant difference) between ITT and PP, ITT findings are discussed hereafter. For pain intensity, participants in the real APA reported a significant decrease in “Worst Pain” and physical function (RMDQ) when compared to participants in the shame group at EOI. Additionally, these changes were maintained at 1-month follow-up compared to participants in the sham APA group.

The proportions of participants in the real APA group who experienced clinically significant, pain intensity decreases expressed as “moderately important” in “worst pain” (decreased ≤30%) at EOI were statistically significant compared to the proportions of participants in the sham APA group at the 1-month follow-up assessment (*P* < 0.0001 and *P* < 0.0001, respectively; see Table 5). For the RMDQ, 24% of participants in the real APA group experienced a 27% reduction in symptoms from baseline to EOI and remained at the same improved percentage at 1-month follow-up. In contrast, participants in the sham APA group had 0% reduced symptoms from baseline after completing a 4-week APA treatment and a 2% reduction at 1-month follow-up.

### 3.5. Secondary Outcomes

Table 6 lists other outcomes of the APA treatment for the real and sham APA groups, outcomes at baseline, at EOI and at 1-month follow-up without treatment. Only ITT findings are presented. For Emotional Functioning, participants in the real APA group all reported a decrease in anxiety and depression after 4-week APA from baseline, but only GAD-7 reached statistical significance. GAD-7 changes were also maintained until the conclusion of the 1-month follow-up. For Pain Beliefs, Catastrophizing, and Health-related Quality of Life, these findings were not statistically significant, but they followed the expected direction.

## 4. Discussion

This is the first study to examine the feasibility, safety, and initial treatment effects of a 4-week APA protocol to manage CLBP in an elderly population. Our study findings indicate that APA is feasible for older populations in terms of recruitment, retention, and adherence. In order to examine the effects of APA while considering dropout, we used both ITT and PP analyses. Although the findings of ITT and PP were slightly different, they did not display significant differences, and we, thus, based our discussion on the ITT.

In recruiting participants, we gathered 57 potential participants with minimal advertisement within 9 months. As seven participants (among the 37 who participated in the study) were referred by the participants who had enrolled in the study, older adults seem to be enthusiastic about APA treatment. In terms of retaining participants, the retention rate was 74% (*n* = 14) in the real APA group and 50% (*n* = 9) in the sham APA group. Although the participants had been verbally informed about the study purpose and study procedures (e.g., the need to travel to the study site six times during the phone screening), participants still needed to read and sign the consent form, complete the baseline assessment, and receive the APA treatment during their first office visit. Travel may be difficult for our older participants who are generally less mobile and need the transportation help. Participants who returned to receive the second week of treatment in both the real and sham APA groups tended to complete the remainder of the study assessment. Thus, we believe that participants who dropped out may not have had enough time to consider their commitment to the study when they came for the first office visit. In any future studies, we would mail the consent form to the participants prior to the consent process obtained during the face-to face-interview to allow them ample time to review the details of the study before agreeing to participate. In addition, providing transportation is very important for this population and would have helped retention. For adherence, participants exhibited an 85% or greater adherence rate throughout the 4-week APA for both the real and sham APA groups.

APA is a relatively safe treatment as the minimal adverse effects were reported by the participants. Participants indicated the adverse effects were bearable compared to their CLBP. No participant dropped out from the study due to the adverse effects of APA. The most common side effect was itchiness of the ear (*n* = 7 [19%] of 37 total participants). No participants dropped out from the study due to the itchiness, which implies that itchiness was a tolerable side effect. During data collection, participants also complained of sleep disturbance due to the seeds if they slept on their side that was being treated with APA. In this situation, they needed to find a comfortable position in which to sleep. Collectively, the findings indicate that APA is relatively safe for treating CLBP in an older population, especially relative to other treatment options available such as opiates or surgery. In future studies, it would be important to inform participants about these possible adverse effects during the consent process and reemphasize them before the APA treatments.

The use of APA for pain reduction and the improvement of physical functioning are promising. Reductions of pain intensity and improvement of physical functioning and pain quality reported at completion of the 4-week APA treatment from baseline in the real APA group were all statistically greater than those in the sham APA group. In addition to statistical significance, clinically important differences for the primary outcomes (including pain intensity and interferences) were also recorded. While these findings are promising, the interpretation and extrapolation of the study findings are limited by several factors that include (1) small sample size due to the nature of the pilot data, (2) unblinding of the primary investigator as the treating clinician, (3) high dropout for the participants in the sham group, and (4) short-term follow-up (only 1-month follow-up without treatment). These shortcomings will be addressed in a future study.

In this pilot study, the primary outcomes show promising evidence for APA effectiveness. Participants in the real APA treatment group reported statistically significant improvements in pain intensity and physical functioning, which included a clinically significant improvement of 30% or greater. Moreover, participants in the real APA group also reported statistically significant improvements in pain quality compared to those in the sham APA group after completion of 4-week APA from baseline. A further study is needed to replicate and expand the current study design. A large-scale randomized clinical trial is needed to determine the efficacy of APA treatment for CLBP that considers other confounding variables, which include the effects of treatment variables (i.e., point specificity and stimulation), placebo effects, and patient expectations of treatment outcomes.

In this study, point specificity is addressed through two-group design using seeds taped onto both real and sham acupoints. The significant changes in pain intensity and physical functioning after completion of the 4-week APA from baseline suggest that we achieved a credible sham comparison. This is consistent with the conclusions of the meta-analysis [24], which suggests that the effects of auricular therapy are indeed acupoint specific. Despite this, the current study design did not include another important variable of treatment: form of stimulation. In other words, we do not know whether the taped-on seeds alone without any pressure would be effective for treating CLBP. To address this issue, one possible study design would be to include another control group (having only tape placed on the real acupoints that would not be pressed by the therapist or participants) to identify whether or not the form of stimulation would have the same treatment effects. Placebo effects, including relationship with therapist and expectancy effects, also must be controlled in the future study to better determine the specific APA effects for the treatment of CLBP.

## 5. Conclusions

In summary, our preliminary findings have shown that APA is both feasible and safe for elderly individuals with CLBP to practice at home. We believe that replicating this study in a randomized clinical trial with a larger sample size is the next step to confirm the efficacy of APA for treating CLBP in older populations.

## Figures and Tables

**Table 1:** Outcome measures used in this study.

	Items	Score range	Internal consistency (Cronbach's *α*)
Pain intensity			
Worst pain (BPI-sf)	1	0–10	n/a
Overall pain intesnity (BPI-sf)	4	0–40	0.69
Pain severity (MPI-s)	2	0–12	0.86
Physical functionning			
RMDQ	24	0–24	0.92
Interference (BPI-sf)	7	0–28	0.96
Interference (MPI-s)	2	0–14	0.89
Pain quality (MPQ-sf)			
Sensory	11	0–44	0.74
Affect	5	0–20	0.82
Total MPQ-sf	16	0–64	0.82
Other dimensions related to pain			
Affective distress related to pain (MPI-s)	2	0–8	0.77
Life control related to pain (MPI-s)	2	0–8	0.90
Emotional functionning			
Anxiety (PROMS)	8	8–40	0.96
Depression (PROMS)	8	8–40	0.94
Generalized anxiety Disorder 7 (GAD-7)	7	0–21	0.80
Pain beliefs (fear avoidance)			
Fear-physical	4	0–24	0.88
Fear-work	7	0–42	0.85
Catastrophizing (PCS)			
Rumination	4	0–16	0.86
Maginification	3	0–12	0.61
Helplessness	6	0–18	0.91
Health related quality of life			
Physical	7	7–28	0.76
Psychological	6	6–24	0.73
Social	3	3–12	0.74
Environment	8	8–32	0.85

BPI-sf: brief pain inventory short form.

MPI-s: multidimensional pain inventory screening.

MPQ-sf: McGill Pain Questionnaire Short Form.

RMDQ: Roland-Morris Disability Questionnaire.

PCS: The Pain and Catastrophizing Scale.

PROMIS: patient-reported outcomes measurement information system.

**Table 2:** Demographic characteristics of the participants.

Variable	Treatment condition		*P* value
	Real *N* (%)	Sham *N* (%)	
Age			
Mean (SD)	70.6 (4.67)	76.7 (7.00)	0.01
Range	65–82	66–90	
Gender			
Male	4 (21%)	7 (39%)	0.24
Female	15 (79%)	11 (61%)	
Race/ethnicity			
White	17 (89%)	15 (83%)	0.59
Black/african american	2 (11%)	3 (17%)	
Marital status∗			
Currently married	10 (52%)	7 (39%)	0.86
Divorced	3 (16%)	3 (17%)	
Widowed	5 (23%)	5 (28%)	
Never married	1 (5%)	2 (11%)	
Employment situation∗			
Working (full time)	2 (11%)	2 (11%)	0.76
Working (part time)	2 (11%)	1 (5%)	
Not employed	1 (5%)	3 (17%)	
Retired	14 (73%)	11 (61%)	
Education level∗			
<8th grade	1 (5%)	0 (0%)	0.75
8th to 11th grade	0 (0%)	1 (5%)	
High school	5 (26%)	4 (22%)	
Technical or vocational school	1 (5%)	2 (11%)	
College and graduate	12 (64%)	10 (56%)	
Estimated income before taxes∗			
Less than $10,000	2 (11%)	5 (28%)	0.07
$10,000 to $19,999	2 (11%)	4 (22%)	
$20,000 to $39,999	7 (37%)	1 (5%)	
$40,000 to $59,000	4 (21%)	1 (5%)	
$60,000 to $100,000	1 (5%)	3 (17%)	
More than $100,000	0 (0%)	1 (5%)	
Causes of back pain			
Osteoporosis	2 (11%)	4 (22%)	0.37
Osteoarthritis	7 (37%)	6 (33%)	0.72
Scoliosis	4 (21%)	3 (17%)	0.67
Disk herniation	4 (21%)	2 (11%)	0.37
Spinal stenosis	6 (32%)	9 (50%)	0.30
Spondylitis	1 (5%)	0 (0%)	0.31
Spondylosis	2 (11%)	0 (0%)	0.15
Vertebral fracture	1 (5%)	1 (5%)	1.00
Current treatment for back pain∗			
Yes, currently	4 (21%)	5 (28%)	
NOT currently, but past	11 (58%)	9 (50%)	0.82
Never	3 (16%)	2 (11%)	
Current pain medication use			
Yes	10 (53%)	7 (39%)	0.40
No	9 (47%)	11 (61%)	
Current sleep medication use			
Yes	3 (16%)	2 (11%)	0.68
No	16 (84%)	16 (89%)	
Satisfied with current treatment∗			
Yes	9 (47%)	9 (50%)	0.23
No	4 (21%)	1 (5%)	

*n varies due to missing data.

**Table 3:** Adverse effects of APA treatment^a^.

	1st Week *n* (%)	2nd Week *n* (%)	3rd Week *n* (%)	4th Week *n* (%)
Pain	2 (5.4)	1 (2.7)	2 (5.4)	2 (5.4)
Discomfort	1 (2.7)	1 (2.7)	2 (5.4)	1 (2.7)
Itching	4 (10.8)	7 (18.9)	7 (18.9)	4 (10.8)
Pressure	2 (5.4)	1 (2.7)	0 (0.0)	0 (0.0)
Burning	1 (2.7)	0 (0.0)	0 (0.0)	0 (0.0)
Numbness	0 (0.0)	1 (2.7)	1 (2.7)	1 (2.7)
Tenderness	3 (8.1)	2 (5.4)	1 (2.7)	1 (2.7)
Soreness	2 (5.4)	2 (5.4)	2 (5.4)	1 (2.7)
Tingling	1 (2.7)	0 (0.0)	0 (0.0)	0 (0.0)
Skin irritation	0 (0.0)	0 (0.0)	0 (0.0)	1 (2.7)

^a^Values are presented as number (*n*) and percentage (%).

**Table 4:** Primary outcome measures.

	BL score	Change BL to EOI	Effect size (Cohen's *D*)	*P* value	Change BL to follow-up	Effect size (Cohen's *D*)	*P* value
Worst pain							
Real-ITT	7.32 ± 2.03	−3.00	1.22	<0.01	−3.16	1.28	0.01
Sham-ITT	7.28 ± 1.90	−0.33			−0.06		
Worst pain							
Real-PP	7.14 ± 2.35	−3.21	1.07	0.02	−3.43	1.20	0.01
Sham-PP	6.78 ± 1.72	−0.78			−0.22		
RMDQ							
Real-ITT	11.11 ± 6.11	−2.95	0.92	<0.01	−2.63	0.65	0.05
Sham-ITT	14.11 ± 4.57	−0.11			−0.33		
RMDQ							
Real-PP	11.00 ± 6.67	−3.21	1.10	0.02	−2.79	0.68	0.13
Sham-PP	11.00 ± 3.20	0.33			−0.11		

ITT: intention-to-treat.

PP: per-protocol.

RMDQ: Roland-Morris Disability Questionnaire.

EOI: end of intervention.

BL: baseline.

**Table 5:** Summary statistics for clinically significant change in pain intensity and back-specific disability by treatment groups.

	Treatment response at EOI ↓ ≥ 30% (*n*)	Treatment response at EOI ↓ < 30% (*n*)	Effect size (Odds ratio)	*P* value	Treatment response at follow up ↓ ≥ 30% (*n*)	Treatment response at follow up ↓ < 30% (*n*)	Effect size (Odds ratio)	*P* value
Worst Pain								
Real-ITT	9	10	7.07	<0.01	11	8	10.36	<0.01
Sham-ITT	2	16			2	16		
Real-PP	8	6	2.60	<0.01	10	4	5.08	0.02
Sham-PP	2	7			2	7		
Disability RMDQ								
Real-ITT	9	10	7.97	<0.01	9	10	7.97	<0.01
Sham-ITT	1	17			1	17		
Real-PP	8	6	4.66	0.03	8	6	4.66	<0.01
Sham-PP	1	8			1	8		

ITT: intention-to-treat.

PP: per-protocol.

RMDQ: Roland-Morris Disability Questionnaire.

EOI: end of intervention.

**Table 6:** Other outcomes measures.

	Group	BL mean ± SD	Change BL to EOI	*P* value^†^	Change BL to follow-up	*P* value^†^
Pain quality						
Sensory	Real	17.27 ± 4.69	−7.36	0.02	−7.82	0.01
(MPQ-sf)	Sham	9.63 ± 3.50	−0.25		3.75	
Affect	Real	5.91 ± 4.18	−3.09	0.01	−3.82	0.00
(MPQ-sf)	Sham	3.00 ± 2.00	−0.25		1.00	
Total MPQ-sf	Real	25.73 ± 7.03	−11.64	0.00	−12.82	0.00
	Sham	14.25 ± 4.89	−0.25		4.88	
Affective distress (MPI-s)	Real	2.50 ± 2.24	−0.95	0.13	−1.32	0.13
	Sham	1.63 ± 0.99	−0.19		−0.38	
Life control (MPI-s)	Real	4.68 ± 1.23	0.09	0.89	−0.54	0.89
	Sham	3.63 ± 1.75	0.50		0.56	
Emotional functionning						
Anxiety	Real	17.33 ± 8.97	−3.50	0.29	−3.50	0.29
	Sham	18.44 ± 7.09	−2.88		−1.33	
Depression	Real	15.25 ± 11.09	−3.42	0.71	−4.42	0.71
	Sham	14.11 ± 5.78	−2.55		0.33	
GAD-7	Real	7.00 ± 6.08	−3.82	0.02	−3.36	0.13
	Sham	3.75 ± 3.45	0.13		0.75	
Pain beliefs (fear avoidance)						
Fear-physical	Real	15.79 ± 6.95	−5.29	0.24	−4.79	0.62
	Sham	14.11 ± 5.60	−1.78		−3.55	
Fear-work	Real	13.07 ± 12.90	−2.00	0.82	−2.14	0.82
	Sham	7.89 ± 9.57	−1.22		−1.45	
Catastrophizing (PCS)						
Rumination	Real	6.43 ± 4.88	−2.29	0.28	−2.29	0.28
	Sham	6.44 ± 5.10	−2.22		−2.33	
Maginification	Real	3.79 ± 3.02	−2.15	0.20	−0.93	0.83
	Sham	2.44 ± 1.74	−1.55		−1.00	
Helplessness	Real	8.43 ± 8.16	−4.57	0.67	−4.72	0.21
	Sham	8.00 ± 4.69	−4.89		−2.78	
Health related quality of life						
Physical	Real	13.31 ± 2.57	−0.45	0.14	0.04	0.76
	Sham	11.37 ± 2.51	0.38		0.38	
Psychological	Real	13.14 ± 2.91	0.53	0.48	1.19	0.38
	Sham	12.96 ± 2.00	1.34		0.74	
Social	Real	15.33 ± 3.58	−0.47	0.74	−0.66	0.56
	Sham	12.89 ± 3.20	−0.15		0.15	
Environment	Real	15.50 ± 2.75	0.71	0.05	0.75	0.06
	Sham	15.89 ± 3.08	−0.95		−0.89	

BPI-sf: brief pain inventory short form.

MPI-s: multidimensional pain inventory screening.

MPQ-sf: McGill Pain Questionnaire Short Form.

RMDQ: Roland-Morris Disability Questionnaire.

GAD-7: generalized anxiety disorder 7.

PCS: The Pain and Catastrophizing Scale.

BL: baseline.

EOI: end of intervention.

^†^Between group comparison.
